# Evaluating User Experiences of the Secure Messaging Tool on the Veterans Affairs’ Patient Portal System

**DOI:** 10.2196/jmir.2976

**Published:** 2014-03-06

**Authors:** Jolie N Haun, Jason D Lind, Stephanie L Shimada, Tracey L Martin, Robert M Gosline, Nicole Antinori, Max Stewart, Steven R Simon

**Affiliations:** ^1^Department of Veterans AffairsHSR&D/RR&D Center of Innovation on Disability and Rehabilitation ResearchJames A Haley VA Medical CenterTampa, FLUnited States; ^2^Department of Community & Family HealthUnversity of South Florida, College of Public HealthTampa, FLUnited States; ^3^Department of AnthropologyUniversity of South FloridaTampa, FLUnited States; ^4^Department of Veterans AffairsCenter for Healthcare Organization and Implementation Research (CHOIR) and National eHealth Quality Enhancement Research Initiative (QUERI) Coordinating CenterEdith Nourse Rogers Memorial VA HospitalBedford, MAUnited States; ^5^Department of Health Policy and ManagementBoston University School of Public HealthBoston, MAUnited States; ^6^Division of Health Informatics and Implementation ScienceDepartment of Quantitative Health SciencesUniversity of Massachusetts Medical SchoolWorcester, MAUnited States; ^7^Department of Veterans AffairsVA New England Health Care SystemBedford, MAUnited States; ^8^Department of Veterans AffairsJames A Haley VA Medical CenterTampa, FLUnited States; ^9^Department of Veterans AffairsCenter for Healthcare Organization and Implementation Research, Section of General Internal MedicineVA Boston Healthcare SystemBoston, MAUnited States

**Keywords:** veterans, secure messaging, patient-provider communication, Department of Veterans Affairs, usability testing, mixed methods, patient-centered care

## Abstract

**Background:**

The United States Department of Veterans Affairs has implemented an electronic asynchronous “Secure Messaging” tool within a Web-based patient portal (ie, My Health*e*Vet) to support patient-provider communication. This electronic resource promotes continuous and coordinated patient-centered care, but to date little research has evaluated patients’ experiences and preferences for using Secure Messaging.

**Objective:**

The objectives of this mixed-methods study were to (1) characterize veterans’ experiences using Secure Messaging in the My Health*e*Vet portal over a 3-month period, including system usability, (2) identify barriers to and facilitators of use, and (3) describe strategies to support veterans’ use of Secure Messaging.

**Methods:**

We recruited 33 veterans who had access to and had previously used the portal’s Secure Messaging tool. We used a combination of in-depth interviews, face-to-face user-testing, review of transmitted secure messages between veterans and staff, and telephone interviews three months following initial contact. We assessed participants’ computer and health literacy during initial and follow-up interviews. We used a content-analysis approach to identify dominant themes in the qualitative data. We compared inferences from each of the data sources (interviews, user-testing, and message review) to identify convergent and divergent data trends.

**Results:**

The majority of veterans (27/33, 82%) reported being satisfied with Secure Messaging at initial interview; satisfaction ratings increased to 97% (31/32, 1 missing) during follow-up interviews. Veterans noted Secure Messaging to be useful for communicating with their primary care team to manage health care needs (eg, health-related questions, test requests and results, medication refills and questions, managing appointments). Four domains emerged from interviews: (1) perceived benefits of using Secure Messaging, (2) barriers to using Secure Messaging, (3) facilitators for using Secure Messaging, and (4) suggestions for improving Secure Messaging. Veterans identified and demonstrated impediments to successful system usage that can be addressed with education, skill building, and system modifications. Analysis of secure message content data provided insights to reasons for use that were not disclosed by participants during interviews, specifically sensitive health topics such as erectile dysfunction and sexually transmitted disease inquiries.

**Conclusions:**

Veterans perceive Secure Messaging in the My Health*e*Vet patient portal as a useful tool for communicating with health care teams. However, to maximize sustained utilization of Secure Messaging, marketing, education, skill building, and system modifications are needed. Data from this study can inform a large-scale quantitative assessment of Secure Messaging users’ experiences in a representative sample to validate qualitative findings.

## Introduction

The Institute of Medicine (IOM) has identified patient-provider communication as a central component to improving quality of care and patient outcomes [1]. My Health*e*Vet is the Department of Veterans Affairs’ (VA) online patient portal and personal health record designed for veterans, active duty service members, and their dependents and caregivers. My Health*e*Vet provides veterans with tools (eg, Blue Button, VA immunization records, laboratory test results, prescription refills, VA appointments) to make informed decisions and manage their health care. “Secure Messaging” is an email-like electronic resource within My Health*e*Vet designed to promote continuity of patient-provider communication [2-4]. As VA further implements Patient Aligned Care Teams (PACT) as a model of the patient-centered medical home, secure messaging is emerging as a key mechanism of communication between veterans and their health care team members. Successful implementation of secure messaging is therefore a priority not only for VA but also for other health care systems in the United States that strive to adopt principles of the patient-centered medical home. Moreover, outside VA, providers are being incentivized via Stage 2 Meaningful Use requirements (Medicare Electronic Health Records (EHR) Incentive Program) to use secure messaging among at least 5% of their patients to communicate relevant health information [5].

Previous work has demonstrated the utility and value of providing patients access to their electronic health record [6-8]. Patients also value secure messaging to communicate electronically with their providers [2-4]. Effective use of secure messaging can improve patient self-care management, patient engagement, and utilization of health services. In addition to allowing an option for self-care management, this electronic tool holds potential for supporting clinical tasks including medication reconciliation [9]. Secure messaging supports system utilization benefits in addition to perceived benefits by patient and clinical team users. A recent study showed a 7-10% decrease in outpatient visits and a 14% reduction in telephone contacts as a result of secure messaging [10,11]. Houston et al reported that 95% of respondents felt email was a more efficient means of communication with their physicians than the telephone, and 77% noted being able to communicate adequately via email without a face-to-face appointment [4]. Patient use of secure messaging has been associated with improved outcomes for chronic conditions [10,12]. Zhou et al reported in a recent study that within a two-month period there were improvements in care as measured by the Healthcare Effectiveness Data and Information Set (HEDIS) [10]. Patients with diabetes using secure messaging improved on all measures recommended for testing and control of glucose, cholesterol, and blood pressure levels by an average of 2.4-6.5% compared with patients not using secure messaging. In the same study, rates of received health services improved in the secure messaging group compared to the control group [10]. These findings suggest that successful implementation of secure messaging may provide a viable cost-efficient means of patient-provider communication.

Implementing health information technology, such as secure messaging, requires systematic inquiry grounded in implementation science to identify barriers to and facilitators of user adoption and utilization. The Technology Acceptance Model (TAM) [13] and the Theory of Planned Behavior (TPB) [14] have been found to be useful in predicting adoption of technology. While secure messaging has been shown to promote continuous and coordinated patient-centered care, little research has evaluated patients’ experiences with and preferences for using secure messaging. In order to maximize sustained utilization of secure messaging, marketing, education, skill building, and minor system modifications may be needed. Evaluation of secure messaging users’ experiences using the TAM and TPB frameworks can increase our understanding of issues related to access, continuity, and coordination of care for veterans that will support adoption and long-term utilization of Secure Messaging in My Health*e*Vet.

Findings from the Secure Messaging evaluation research will inform efforts to transform care delivery both within and beyond the VA system. Thus, the aims of this study were to (1) characterize veterans’ beliefs, attitudes, and perceptions toward using the Secure Messaging tool, (2) describe the patterns of veterans’ use of Secure Messaging, (3) identify the barriers to and facilitators of using Secure Messaging, and (4) describe strategies for promoting facilitators and overcoming barriers to using Secure Messaging.

## Methods

### Study Design

This prospective descriptive qualitative study used mixed-methods to describe veterans’ experiences using Secure Messaging in the My Health*e*Vet portal. As an implementation study, the underlying objective was to understand veterans’ needs to promote increased access to and sustained utilization of the Secure Messaging tool. A combination of in-depth interviews, user-testing, a 3-month review of transmitted secure messages between veterans and staff, and 3-month follow-up phone interviews was used to characterize veteran Secure Messaging utilization. Demographic data as well as computer and health literacy measurements were collected through survey and in-depth interviews at baseline and 3-month follow-up.

### Setting and Participants

The two-site study was conducted at two large VA Medical Centers (VAMCs): the James A. Haley Veterans' Hospital (Tampa, Florida) and the Veterans Affairs Boston Healthcare System (Boston, Massachusetts). We used administrative data to identify veterans at both VAMCs who had registered for My Health*e*Vet, completed the in-person process of authenticating their identity, and accessed the system to “opt-in” to use Secure Messaging. This approach identified 3926 potential participants at Tampa and 924 at Boston. Next, randomization was used to create contact lists of 120 potential participants from each site list. All 240 potential participants were contacted and screened to be purposively sampled based on their self-reported previous use of Secure Messaging. Participants were recruited for study participation until domain and theme saturation was reached.

Inclusion criteria included veterans who were independent Secure Messaging users, without cognitive impairment that prevented use of a personal computer or the ability to provide informed consent. Based on qualitative sampling methods [15,16], saturation was anticipated to occur between 12 to 15 interviews; an over-recruitment strategy was used at each site to allow for attrition, resulting in 33 total participants. One participant was lost to follow-up for unknown reasons, resulting in a complete dataset of 32 participants. Veterans received up to US$50 for their participation: US$20 for participation in the initial interview and user-testing and an additional US$30 for allowing the researchers unrestricted access to review the content of their secure messages and participation in the 3-month follow-up telephone interview. Participants provided informed consent upon their arrival for the initial face-to-face interview and user-testing. This study was approved and regulated by the VA Central Institutional Review Board.

### Data Collection Instruments

#### Overview

Data were collected using demographic and health literacy surveys, in-depth face-to-face interviews, Secure Messaging usability testing, prospective collection of the content of secure messages, and 3-month follow-up telephone interviews. All data, with exception of the Secure Messaging data, were collected at two time points: during a baseline in-person meeting and during a 3-month follow-up phone interview. Prospective Secure Messaging data were collected between the baseline and 3-month follow-up time points.

#### Participant Surveys and Assessments

During the initial research visit, veterans completed a 13-item demographic survey to ascertain age, gender, race/ethnicity, education level, income level, marital status, computer use, Internet use, My Health*e*Vet use, and Secure Messaging use. Health literacy was assessed using two validated instruments: (1) the Brief Health Literacy Screening Tool (BRIEF), and (2) the Rapid Estimate of Adult Literacy in Medicine (REALM) survey. The BRIEF is a 4-item self-report screening tool to assess health literacy skills [17]. The REALM assesses health literacy by having respondents verbally articulate three columns of 22 health-related terms [18].

Electronic health literacy was also assessed using two instruments: (1) the eHealth Literacy Scale (eHEALS), and (2) the Computer-Email-Web (CEW) Fluency Scale. The eHEALS is a 10-item measure of eHealth literacy developed to measure consumers’ knowledge, comfort, and perceived skills at finding, evaluating, and applying electronic health information to health problems [19]. The CEW Fluency Scale is a 21-item measure of common computer skills [20].

#### Interviews

Face-to-face semi-structured interviews with participants were conducted by an experienced interviewer trained in the social sciences. Interviews focused on participants’ experiences using Secure Messaging. The interview guide was created following the Theory of Planned Behavior (TPB) framework to elicit beliefs and attitudes, subjective norms, perceived behavioral control, and behavioral intention toward Secure Messaging use. Other interview questions were developed based on the Technology Acceptance Model (TAM) and addressed usefulness and ease of use of Secure Messaging. Interviews followed the guide but were open-ended in nature, allowing the interviewer flexibility to ask probing questions and to follow up on interesting topics and user experiences related to Secure Messaging.

Based on the initial interviews, a brief phone interview guide was developed to address Secure Messaging use during the 3-month period after the first interview. These interviews were conducted to assess recent Secure Messaging use: usefulness, expectations, barriers and facilitators, satisfaction, and suggestions for improvement.

#### Secure Messaging User-Testing

In-person Secure Messaging user-testing was conducted to prompt participants to complete a series of tasks they would normally encounter while using Secure Messaging. User tasks included navigating to the My Health*e*Vet site, logging in to Secure Messaging, setting user preferences, checking the Inbox, opening a secure message, opening and reading an attachment, and sending a secure message. Task completion, obstacles, and facilitators were recorded using a checklist, which directly corresponded to the user-testing tasks. Usability testing with each participant was conducted using Morae software [21,22], and allowed for the live, remote observation and video-recording of the user being tested (eg, recording of clicks, keystrokes, and other events) [23]. Participants were asked to “think aloud” and vocalize their thoughts, experiences, feelings, and opinions while interacting with the program as they used the Secure Messaging feature [24,25].

#### Secure Messaging Content

Secure messages were collected, both outgoing and incoming secure messages were collected for each participant over a 3-month period following their provision of informed consent. Data included sender and recipient identification, date and time of delivery, subject header, category of message subject (eg, test, appointment, medication, general), and verbatim content of the secure message text. We examined the quantity of messages, message content, exchange patterns, and timing of inbound and outbound messages between participants and their health care teams. This approach allowed for analysis of authentic user content and patterns to further inform research findings.

### Data Management and Analysis

All data, including interviews and paper-based surveys gathered in this study were stored on a secure VA network. Audio recordings of all interviews were transcribed and subsequently analyzed using ATLAS.ti [26], qualitative data analysis software. Descriptive statistics from veteran surveys were managed using the statistical software suite SPSS version 21 (SPSS IBM, New York, USA). Data from Secure Messaging usability testing were captured using Morae recording software.

We used content analysis methods to analyze all interview data to identify domains and taxonomies related to participants’ experiences using Secure Messaging [15]. We used the semi-structured interview guide to organize and code interview text to develop thematic categories. Categories were grouped into taxonomic relationships and then compared and contrasted across coded categories. Coding schemas were developed by two research team members to create domains and taxonomies and evaluated for inter-rater reliability and credibility. Data were then categorized and interpreted, and barriers and facilitators were identified. Quantitative data were summarized with descriptive statistics to describe sample characteristics. Frequency counts and proportions provided a descriptive overview of the user-testing findings.

## Results

### Participants

A total of 33 participants were recruited, of whom 32 provided complete data. One participant provided initial interview, user-testing, and secure message content data, but could not be reached for the follow-up phone interview.

### Survey and Assessment Findings

The majority of participants were older white males (26/33, 79%) and ranged in age from 27 to 77 years, mean age 59.5 (SD 11.9). All participants had at least a high school education, and 64% (21/33) had an annual income of US$35,001 or more. Demographic characteristics are reported in Table 1.

Though skills varied, the majority of participants had adequate health literacy and eHealth competency skills. Study participants had higher levels of health literacy than the general veteran population [27]. Though comparative studies are not available for this population using these tools, the electronic health literacy scores on the eHEALS and the CEW produced similar findings. Instrument range, sample range, mean, and SDs are illustrated in Table 2.

At baseline, all participants (n=33, 100%) reported using a computer and the Internet more than once a week. Most participants (22/33, 67%) reported using Secure Messaging for at least the past six months (10/33, 30%) or longer (12/33, 36%), while the remaining participants reported using Secure Messaging for less than six months (11/33, 33%). The majority of participants (28/33, 85%) reported using Secure Messaging “at least once a month” (12/33, 36%) or “a few times a year” (16/33, 49%). Most veterans (27/33, 82%) reported being satisfied with Secure Messaging.

**Table 1:** Demographic characteristics of study participants (n=33).

Characteristic		n (%)
**Gender**		
	Male	26 (79)
	Female	7 (21)
**Education**		
	High School	4 (12)
	Some College/Vocational	6 (18)
	Associate Degree	6 (18)
	College Degree	8 (24)
	Graduate Degree	9 (27)
**Ethnicity**		
	Caucasian/White	22 (67)
	African American/Black	5 (15)
	Hispanic/Latino	2 (6)
	American Indian/Alaskan Native	1 (3)
	Unknown/Missing	3 (9)
**Annual income (US$)**		
	$5,000 - $10,000	1 (3)
	$10,001 - $15,000	3 (9)
	$15,001 - $25,000	4 (12)
	$25,001 - $35,000	2 (6)
	$35,001 - $45,000	5 (15)
	More than $45,000	16 (48)
	Missing	2 (6)

**Table 2:** Instrument range, sample range, mean, and standard deviation.

Instrument	Possible range	Sample range	Sample mean	SD
BRIEF^a^	4-20	10-20	17.7	2.5
REALM^b^	0-66	55-66	63.3	2.8
eHEALS^c^	10-50	29-50	42.7	5.7
CEW^d^	18-90	54-90	82.8	10.4

^a^BRIEF: Brief Health Literacy Screening Tool

^b^REALM: Rapid Estimate of Adult Literacy in Medicine Survey

^c^eHEALS: eHealth Literacy Scale

^d^CEW: Computer-Email-Web (CEW) Fluency Scale

### Interview Findings

#### Overview

Qualitative analysis of interviews revealed that veterans valued Secure Messaging based on time saving, data security, and the ease and efficiency of communicating with their health care team. Veterans asserted that Secure Messaging was an excellent alternative to calling the hospital, allowing them to communicate with their primary care team at their convenience (eg, late at night). Veterans reported the top reasons they used Secure Messaging included: (1) general questions, (2) medication refills, (3) appointments, and (4) test results. Veterans also expressed satisfaction with the timely manner of Secure Messaging communication, generally receiving a response from their primary care team within 48 hours. Veterans reported no problems understanding secure message responses from their primary care team members, and few veterans noted being uncomfortable sharing private health information through Secure Messaging. Interview themes emerged in four major domains: (1) perceived benefits of using Secure Messaging, (2) barriers to using Secure Messaging, (3) facilitators of using Secure Messaging, and (4) suggestions for improving Secure Messaging.

#### Perceived Benefits of Using Secure Messaging

This domain focused on benefits related to resource and communication efficiency between veterans and their primary care team, as seen in Table 3. Veterans highlighted the fact that Secure Messaging saved them time and resources by providing them “24/7” access to their primary care team. Veterans noted that, through Secure Messaging, appointments could be made, referrals provided, and prescriptions filled, thus avoiding the frustration of spending hours on the phone or driving long distances to accomplish these tasks face-to-face. Veterans also reported that having 24-hour access to Secure Messaging increased their ability to communicate effectively with their primary care team and that this increased access gave them the ability to send a secure message late at night, instead of waiting to call during business hours. Similarly, veterans indicated that Secure Messaging afforded them the ability and confidence to draft a question to their provider in their own time and without the pressure of having to relay the same question over the phone or in person. Having a written record of Secure Messaging conversations also helped veterans effectively communicate their questions and concerns to their provider.

**Table 3:** Exemplar quotes of perceived benefits of using Secure Messaging.

Theme	Exemplar quotes
Resource efficiency	*It’s [Secure Messaging] immediate and hands-on—you don’t have to wait 6 months for an appointment, you don’t have to go through acute care, you don’t have to go through all this stuff just to get a referral to your primary [care physician]…so it’s contacting the primary immediately, which is great…I think it saves money, ‘cause every time you go to acute care that costs money. So I think it saves money and a lot of time, a lot of wasted time, you know.*
	*That direct communication without having to stop what I was doing to go see [my primary] and try to make an appointment or get in. I can talk bluntly [on Secure Messaging] you know, just like we were face-to-face. I love that, because there were some really personal things going on, you know, with me and my body and I was like, give me some advice on this, and she would just email right away…*
Communication efficiency	*I like to do most of my studying at night and if I happen to think that I got to re-order this prescription, I just get on [Secure Messaging] and do it. It’s 24/7 you know, and the next thing I know I got it [prescription] within a week…It doesn’t tie up personnel at the VA. It just makes life easier.*
	*In Secure Messaging, you can narrate [your message] very, very precisely and have it understood by the clinical team that reads your message and it’s a lot better than someone just answering the telephone and then try to decide how your call should be routed.*

#### Perceived Barriers to Using Secure Messaging

This domain encompassed issues related to initiation and knowledge barriers, privacy and security issues, prohibited personal expression, and clinician resistance. These themes included not knowing how to register and initiate the authentication process required to use Secure Messaging, not being able to locate the link within My Health*e*Vet to access the Secure Messaging feature, and not fully understanding the circumstances and situations in which they should use the Secure Messaging tool. These reported barriers are presented in Table 4.

Other barriers included confusion regarding who, among the primary care team, receives their secure messages. For example, some participants reported “learning” that their messages were not going directly to their primary care physician, and felt uncomfortable with the fact that multiple members of their primary care team had access to their secure messages. As a result, these veterans reported being disappointed and indicated that having multiple team members reading their messages would affect the type of health information they would include in future secure messages. Others reported learning that their primary care team discouraged them from sending personal non-health-related information.

Surprisingly, veterans noted VA staff resistance to Secure Messaging use as a barrier to their use of the tool. Several veterans cited having initiated contact with a specialty clinic, pharmacy, or primary care provider through Secure Messaging only to have the clinic attempt to respond to the veteran by telephone, rather than replying via Secure Messaging. Other veterans reported that when they asked their specialist if they could contact them through Secure Messaging, they were told to call the clinic instead. These veterans perceived that staff members were avoiding Secure Messaging in favor of traditional methods of communication.

**Table 4:** Exemplar quotes of barriers to using Secure Messaging.

Theme	Exemplar quotes
Initiation and knowledge barriers	*The authentication process to use [Secure Messaging] is too cumbersome. I feel like if I can come in and see a doctor without having to do all this and the doctor knows it’s me. I mean, this seems like it ought to be something that could be done in your doctor’s office and not some other way. There has to be an easier way to do it.*
	*I would say [Secure Messaging] is a good idea. I’m not 100% sure what you’re supposed to use it for. The site doesn’t tell you so I’m never sure if I’m supposed to ask medical questions or just, hey, can I make an appointment, or hey, can I get a refill. I don’t know how in-depth you’re supposed to get with your provider.*
Privacy and security issues (personal health information)	*I think Secure Messaging has great potential but it just has to be explained…they have to stop letting you think you’re talking to your doctor somehow, it has to be a little clearer…it’s misleading to say you’re sending a message directly to your primary care provider.*
	*I would say it’s an indirect way of getting in touch with your doctor, and it’s open to who knows how many people in the system. I get responses from a lot of people, sometimes the call center even. It’s disconcerting…when I first started using Secure Messaging, it would go directly to [my primary physician] although everybody in the clinic gets them for some reason.*
Prohibited personal expression	*I’ve just made my messages more brief. Instead of sending a clinical message and then tagging on a personal message, I don’t tag on a personal message anymore.*
Clinician resistance	*There’s another specialty clinic I went to not long ago and the [specialist] told me at this point in your treatment I want you to call me and tell me this. He said, ‘I probably won’t answer the phone, so leave a message.’ I said, ‘Can I just use Secure Messaging?’ And he said, ‘No, no, I don’t use that, I don’t want to have an inbox with a thousand messages’.*

#### Facilitators of Using Secure Messaging

This domain included two major categories of facilitators including convenience and Secure Messaging user-friendly features. Participants reported conveniently communicating with their primary care team and getting responses and results. Other facilitators of using Secure Messaging included user-friendly features such as message notification (ie, getting a message via a non-VA email account indicating that there was a new message to be viewed in the Secure Messaging Inbox), dropdown menus, and folders for organizing received and sent messages. Veterans expressed the ways in which these features enhanced their ability to use and manage their secure messages effectively. These reported facilitators are illustrated in Table 5.

**Table 5:** Exemplar quotes of facilitators of using Secure Messaging.

Theme	Exemplar quotes
Convenience	*I was taking nicotine patches, nicotine gum, and they expire quickly, and, if I look at my med history, I’ll see that it’s expired and that I’m not scheduled to see [my primary] to order more. So I would send a secure message stating that I’ve ran out of my nicotine gum…and they came in the mail. And I just love that! I love that, whereas you can use Secure Messaging like that instead of getting on the phone and trying to describe it and telling them your last four of your Social Security Number.*
	*The great thing is, when you get a response from your [primary care] team, you also get a notification in your private email letting you know you have a new Secure Message. So at least I know somebody replied back and that’s a feature that I believe is, is working great, because you know somebody answered you. That way you know, you got to go back and log on and get into your Secure Messaging.*
Secure Messaging user-friendly features	*One feature that I use is you can create more folders, that you can save and divide your messages from your primary doctors, or create another one for your nurse, or your team, or by illness. I have some friends that divided their [secure messages] by illness, because sometimes you’re requesting information about a prescription and you can keep all that in a folder…I try to keep my Inbox clean.*
	*To be able to [use] the dropdown box where I can see my primary doctor, and when I click in her email address and then I can put my message in the area where I’m supposed to type it in and hit send. It’s very simple, you know, it’s very simple. Then I go back and check it in the Inbox I’ll have a message and, uh, just read it…*

#### Suggestions for Improving Secure Messaging

Suggestions for improving Secure Messaging encompassed a broad array of system characteristics and approaches to engaging veterans. Veterans suggested the need for enhancements in the following areas: ease of navigation and use; available features; user interface and visual appearance of the on-screen content; ease of access and log-in; and awareness, education, and marketing (see Table 6). To improve ease of use, participants stated the need for a clearer navigation path to get from the main My Health*e*Vet site to the Secure Messaging feature. Currently, veterans have the option to activate a setting to provide non-VA email notification of Secure Messaging activity; veterans suggested setting the default preference setting for this notification to occur, thereby requiring veterans to change settings to deactivate it. Many veterans reported being unaware of the availability of the message notification feature, yet found it extremely useful once they learned about it and changed their setting preferences to enable it. In addition to simplifying the message notification feature, standard email features were commonly requested by veterans including a print option, spell check, formatting tools, and a message receipt system (ie, an automated reply message stating that their message was received by the intended recipient). Other ease-of-use suggestions included improvements for individuals with visual impairment, such as incorporating larger print/font and icons or images, rather than text, to guide the user; changing the amount of information presented per screen, or otherwise adjusting the display characteristics, to eliminate the need for scrolling; and ensuring key elements such as tabs and icons are clearly visible without magnification. Another suggested improvement was for the veterans’ primary care team members to identify themselves when responding to secure messages. Suggested identifiers included health team members’ names, photos, clinical role, and credentials. This improvement would help to eliminate confusion regarding which individual member among their primary care team was responding to their secure messages.

To increase usefulness, Secure Messaging respondents most commonly reported a strong desire to access specialty clinics via Secure Messaging, especially clinics where they are current patients. Veterans expressed frustration about not having access to some specialists and having to revert to traditional methods of communication. Others cited redundancy in having to ask their primary care physician to facilitate communication with their specialist.

Other suggestions for innovation and improvement included voice/image options (eg, Web cam, live chat), a Secure Messaging application for mobile devices (ie, a smartphone app), separate Secure Messaging log-out from My Health*e*Vet log-out, and the ability to import/attach information from Blue Button (a single electronic file that contains all available personal health information) and other My Health*e*Vet features (eg, test results) into their secure messages when communicating with their VA health care team.

In addition to suggestions to improve ease of use and usefulness, participants reported a need for increasing awareness, education, and instruction about Secure Messaging and My Health*e*Vet. Participant comments also highlighted a need for promotional strategies to facilitate veteran awareness and adoption of Secure Messaging, as represented in Table 6.

**Table 6:** Exemplar quotes of suggestions to improve Secure Messaging.

Theme	Exemplar quotes
Improve ease of navigation and use	*It’s not so easy to get that [Secure Messaging] page where you first start doing it ‘cause it seems like every time I do it I have to, I don’t know, like I end up “Googling” My Health*e*Vet and then I have to look and try and find it and then I have click here and then I have to…I don’t know [laughter].*
	*So it’s hard to find what I’m looking for because as a patient what I’m looking for is, you know, a priority. You gotta figure most patients are like me and they would want appointments and, uh, appointments, medications, and possibly talk to the doctor, but you got all this other stuff, uh, that is competing for your attention.*
	*As it is built right now, I think it keeps me from using it and so it would have to streamline, you know, for me to [use]…have to make it user customized…*
Features	*What I would like to see in the near future, would like to see that Secure Messaging, going probably in some kind of an application in the cell phone, like they’ve been doing, like with the PTSD program. They have some kind of application…but will be great, you know, using the cell phone and having application that can connect you directly. That will be great.*
	*A Web cam, you know what I mean. You could probably do it with that if you could adjust it… you could click on it and see what it looks like and either gives me a decision of what it or, you know, a picture or whatever.*
Screen visualization	*Uh, the Secure Messaging [button] is way up in the right hand corner off the screen and I think it should be more in a more recognizable place, ‘cause if you didn’t know it was there you wouldn’t know it’s there, so I would think to place the button in a more visual place on the, uh, screen, on the window.*
Access to specialty clinics	*Being able to secure message other clinics that I receive care in if there’s other doctors that I see. I see doctors in the stroke clinic, I see doctors in the MOVE clinic, the weight management clinic, um, so I think it would be nice if I could communicate with weight management clinic and the stroke clinic.*
Awareness, education, marketing	*I think that they should have some kind of tutorial, to show every veteran [Secure Messaging] uses so at least everybody would be knowledgeable. Once you see the home page of My Health*e*Vet, you think it’s too cumbersome or too much to get involved…people don’t want to go any further than refills. I think there should be a way on the main page to give them a tutorial on how to use [Secure Messaging] to make it useful to each and every vet.*
	*You don’t get any notification on your email. For instance, like, my email is Hotmail so I don’t get any notification telling me that ‘hey, you’ve got a message on your Secure Messaging, VA has sent you a secure message’ so I don’t get any of that feedback because I’m only on the SM when I need to be…*
	*I don’t see any problems with [Secure Messaging] so I wouldn’t change anything. It’s just getting the information out to the veterans to let them know that there is a convenient and secure way to communicate with your primary.*
	*For me, I honestly, I probably wouldn’t drive all the way to the hospital to go to an education class on [Secure Messaging], but if they would put the same education video on the website, I might watch it so I don’t have to learn through trial and error.*
	*I especially like to read, so if they would just provide information on the website as far as a short piece about how [Secure Messaging] works, what are the benefits, and how to use it, I would definitely read it. I’m not a big fan of paper so I would definitely be happy with something that was online.*

### Follow-Up Interview Findings

All 32 veterans who completed the follow-up interview reported using a computer/Internet more than once a week. Most veterans (84%, 27/32) reported using Secure Messaging “at least once a month”, compared with 36% (12/33) upon initial survey. Almost all veterans (97%, 31/32, 1 missing) reported being satisfied with Secure Messaging. In general, follow-up data mirrored responses to initial (baseline) assessment. The BRIEF (*r*=.51, *P*<.003), CEW (*r*=.61, *P*<.001), and eHEALS (*r*=.58, *P*<.001) were significantly correlated from baseline to 3-month follow-up. Changes in Secure Messaging use in 3-month reports included higher utilization since initial assessment. Those who reported not using the system generally indicated that they had no need to contact their health care team via Secure Messaging during the 3-month time period.

Follow-up interview themes addressed participants’ increased Secure Messaging utilization, learning about Secure Messaging through interviews, and self-care management. A minority of participants cited learning more about Secure Messaging during their initial interview, particularly about reasons for using Secure Messaging and different features such as the folders and the option to receive notification via personal (non-VA) email when a secure message is received. Veterans also mentioned engaging in Secure Messaging more often as a result of having participated in the study, citing greater confidence and understanding of the Secure Messaging interface. A few veterans even indicated that they increased their motivation to improve how they manage their own health care as a result of learning more about Secure Messaging through participating in the study. These comments are reflected in Table 7.

**Table 7:** Exemplar quotes from follow-up interviews.

Theme	Exemplar quotes
Increased Secure Messaging utilization	*I used to use [Secure Messaging] in the past and, uh, it wasn’t as frequently as I do now, based on the fact that I know it’s there and so that’s my first course of action. When I’m thinking about trying to get anything or get answers from my physician, I go through Secure Messaging first thing right off the bat.*
	*Yeah, because I really hadn’t used it much prior to our meeting and, you know, I’ve made a conscious effort of using it versus calling, yes.*
Learning about Secure Messaging through interviews	*I’m impressed. It does make it easier to be notified of a message, uh, especially to be notified of appointments that have been scheduled…and as I mentioned I hadn’t gone that deep into preference, but I know now that I can do this at home with my preferences.*
	*I didn’t know about the draft feature before the interview. Now I can save a draft.*
	*Really, I’ve never thought about user preferences, I just never thought that, if I had an ongoing communication with my primary…I think I would change my user [preferences] whereas right now it’s not set.*
	*I didn’t even know [user preferences] was there. When I go home, I’ll look.*
	*I really hadn’t looked at these dropdown boxes, which make it simple, you know.*
Self-care management	*Yes, and I’m finding that this is a good tool. I’m beginning to recognize that, in the long term, this is going to be beneficial to me especially…now I’m getting old and if I can’t speak, my wife’s gonna have to speak for me with all this information in front and we’ll be able to do that.*
	*I think that it’s helped me become more involved in my health care. Just knowing that I have that opportunity to ask a question and I don’t have to schedule an appointment to ask it. If I do have that question, I’m gonna ask versus not asking it so I can be a little more proactive in my health care versus [waiting] until something happens and then going in and trying to figure out what’s wrong at that point.*

### User-Testing Findings

**Figure 1:**
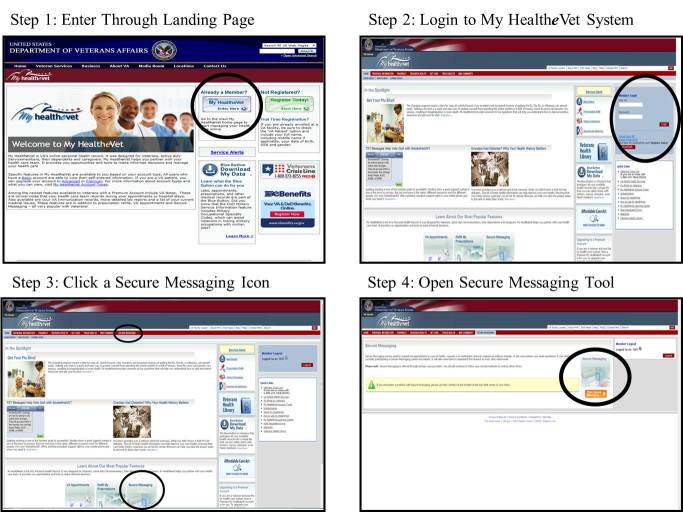
Four-step process for logging in to Secure Messaging.

**Figure 2:**
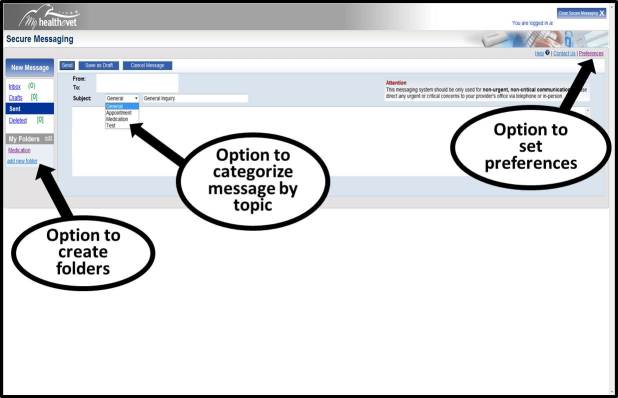
Folder, subject categorization, and preferences features in Secure Messaging.

Though participants were able to complete most tasks necessary to use Secure Messaging, user-testing findings indicated potential opportunities for improving usability, including navigation of the My Health*e*Vet website, setting user preferences, categorizing message subject headings (Secure Messaging users can access a dropdown menu to populate the subject line of their messages with one of four predefined categories, ie, general, appointment, medication, test result inquiry, to indicate the purpose of the message). For example, it currently takes four steps to access Secure Messaging; participants suggested that fewer steps or a separate log-in for Secure Messaging (distinct from the My Health*e*Vet portal), would support easier navigation access to the messaging tool (see Figure 1). Additionally, several features were not easily accessible and sometimes unknown to participants, including setting user preferences and categorizing message subject (see Figure 2). However, opening folders and navigating through folders did not pose issues for participants during the user-testing (see Figure 2). User-testing findings are illustrated in Table 8.

**Table 8:** User-testing findings (n=33).

Task	Able to complete task n (%)	Completed task with difficulty n (%)	Did not complete task n (%)
Navigate to site	21 (64)	10 (31)	2 (6)
Log in to site	30 (91)	2 (6)	1 (3)
Set “User Preferences” option	23 (70)	8 (25)	2 (6)
Check Inbox	33 (100)	0 (0)	0 (0)
Use links within Secure Messaging	33 (100)	0 (0)	0 (0)
Open secure message	33 (100)	0 (0)	0 (0)
Open attachment	33 (100)	0 (0)	0 (0)
Send secure message	30 (94)	3 (9)	0 (0)
Choose recipients for secure message	32 (97)	0 (0)	1 (3)
Categorize message subject	9 (28)	0 (0)	24 (73)
Formulate subject header	18 (55)	0 (0)	15 (47)
Formulate a secure message	33 (100)	0 (0)	0 (0)

### Secure Messaging Data Content Findings

Data were collected for 33 participants during the 3-month review period, of whom 18 (55%) sent a total of 66 secure message threads (series of messages from one original message). Three of the 18 participants’ messages were simply to test the system and did not include any substantive content, and 15 participants (45%) did not use Secure Messaging during the 3-month timeframe, other than when conducting the user-testing on the test account. Nearly all of the message threads (62/66, 94%) were categorized in one of four categories (ie, general, appointment, medication, test) by senders to indicate the message topic. Though the message content matched the selected category in messages sent by secure message senders, due to the generic nature of the categories, “general” was over-used to address all topics, including those represented by the other three categories (ie, appointment, medication, test). Secure message content topics are illustrated by veteran-selected category in Table 9.

A total of 50 (76%) of the 66 veteran-initiated secure messages received responses from their health care team members. Several messages sent by veterans did not result in a response from the health care team; however, some messages revealed that a clinical response had occurred through other mechanisms (eg, veteran sent secure messages thanking team members for a call). For those messages that did receive a reply, response times from VA team members ranged from 8 minutes to 136 hours (>5 days). The expectation for response time, based on VA guidelines, is 3 federal business days after the original secure message is sent.

**Table 9:** Secure Messaging content topic by veteran-selected category.

Category	n	Secure Messaging content topic
General	36	condition management/report, specialty/procedure request, correspondence request, medication refill request, test results, appointment requests, treatment/appointment follow-up (2 messages sent to check if previous messages were received; 1 to report of being removed from team on the secure messaging recipient list)
Appointment	15	confirmations, cancellations, specialty appointment requests
Medication	10	refill requests, medication inquiries
Test	1	test request

## Discussion

### Principal Findings

We conducted a multi-method study of veterans’ use of Secure Messaging, an email-like communication tool embedded in the Department of Veterans Affairs’ online personal health record and patient portal, My Health*e*Vet. We found that the majority of veterans who participated in this study were satisfied with Secure Messaging, reported it to be a useful tool for managing their health care, and generally demonstrated facility using its features. Perceived benefits of Secure Messaging most commonly related to the convenience of communicating with their primary care team. The ability to avoid telephone triage, to send messages at a time of their choosing, and to edit messages before sending them to their primary care teams were strong motivators for continued Secure Messaging use. Similar findings were reported in another study that found patients preferred secure messaging over phone calls to communicate their health care needs [28].

Using TAM and TPB, we prompted participants to identify ways to improve ease of use and make system changes to promote their sustained utilization. Although participants were generally able to complete most of the user-testing tasks with ease, they reported some barriers to use, specifically opportunities for improved usability related to navigating the My Health*e*Vet site, setting user preferences, categorizing messages, and formulating subject headers. Veterans suggested improvements to the system to overcome barriers to use, such as changing the default settings of preferences so that veterans would automatically receive regular email notification when they received a secure message on My Health*e*Vet, and adding more dropdown menus to guide veterans with a set of options populating the subject line of their messages. Our study highlighted a need for promotional strategies, instructional interventions, and aligning expectations for use to support Secure Messaging and My Health*e*Vet utilization across the VA [29].

Though participants reported several benefits and intention to continue Secure Messaging use, barriers to use were also reported. Participants reported perceived resistance from their clinical team members as a barrier to continued use. These results echo a similar study that found physicians consistently preferred traditional methods of communication with patients including face-to-face, telephone, and written communication [30].

Sample characteristics, including educational and income levels as well as eHealth and health literacy levels, were not typical of the general veteran population; however, these findings are consistent with existing literature, which suggests that eHealth users tend to have higher levels of eHealth and health literacy, as well as educational and socioeconomic status, than the general population. As a result, this sample is representative of those more likely to be eHealth users [31-34].

The “learning effect” (eg, learning about features), reported by veterans during face-to-face interviews, indicates the need for the provision of user training. A considerable number of veterans articulated the misperception that only their primary care physician would receive and view their secure messages. Moreover, veterans expressed some degree of betrayal and dissatisfaction when they learned, whether through our study or otherwise, that other members of their health care team could review and respond to their messages. These observations indicate a need to address veterans’ misperceptions and a possible role for enhanced education of and disclosure to veterans about how their clinical team receives and responds to the veterans’ messages. Our findings reveal a need for improved instruction when veterans begin using Secure Messaging to improve uptake, utilization, and sustained use.

Though our results from questionnaires, interviews, user-testing, and review of message content were generally convergent and internally consistent, some data sources indicated discrepancy between veterans’ reports and objective data collection. For instance, more than 80% (27/33) of participants reported using Secure Messaging at least once in the past 3 months, but Secure Messaging content analysis indicated only 55% (18/33) of veteran participants sent messages during that time window. This discrepancy may be result of recall bias or social desirability bias. Another possible explanation for this discrepancy is participants’ confusion between the My Health*e*Vet portal and Secure Messaging tool, such that veterans may have used My Health*e*Vet but not Secure Messaging. Veterans who did not use the system had not perceived a need to communicate at all with their health care team during that time period. This reason for non-use has been provided by participants in previous research [29]. Similarly, the majority of veterans reported receiving Secure Messaging responses within 24-48 hours; however, review of the Secure Messaging content suggested that response times ranged anywhere between 8 minutes to 136 hours (>5 days). Analysis of secure message content data also revealed that veterans sent messages to inquire about sensitive topics, such as sexually transmitted diseases (STDs) and erectile dysfunction (ED), and that these topics were not revealed in interviews. These observations underscore the value of using mixed methods to characterize the uses of Secure Messaging that could not be gleaned from veterans’ self-reports alone. Similarly, face-to-face interviews provided in-depth perspective into how veterans perceive and experience Secure Messaging, while observational data from usability testing revealed how veterans actually interact with the Secure Messaging platform in a “real-world” setting. Multiple datasets also allowed the research team to compare and contrast veterans’ reports with objective Secure Messaging data sources, providing a more thorough and textured understanding of veterans’ experiences when using Secure Messaging.

As health care evolves from a reactive, episodic disease-based paradigm to a preventative continuous health model, large health care systems such as VA will require the integration of electronic health resources to promote continuity and increase communication and workflow efficiency. Consumer adoption and sustained utilization are necessary to leverage these tools and their benefits to capacity. The science of marketing, implementing, disseminating, and sustaining Secure Messaging and other patient-facing eHealth technologies has not yet been perfected. Understanding consumer needs is central to intervening and remediating any barriers to adoption and sustained use of Secure Messaging. Both qualitative and quantitative methods to understand veterans’ and clinicians’ experiences and perceptions of Secure Messaging use are needed to overcome barriers and promote facilitators. This study’s exploratory findings provide a comprehensive framework for future evaluation of Secure Messaging use in a large-scale representative veteran sample.

### Limitations

The mixed methods used in this study with users and non-users of Secure Messaging provided a rich dataset that resulted in a comprehensive perspective of veterans’ experiences using Secure Messaging. Though this study yielded valuable data to inform marketing, education, and system-based changes to improve awareness, adoption, and sustained utilization of Secure Messaging, limitations should be noted when interpreting findings. First, although our sample size was comparable to other qualitative mixed-methods studies [35], these results may not be generalizable to the general veteran patient population. This limitation is particularly salient in this study due to the relatively high levels of education and health literacy among study participants. However, this sample was purposively recruited to represent the perspective of veterans with Secure Messaging access and theoretical saturation was reached. Second, although our sample included veterans who did not use Secure Messaging during the 3-month data collection period, we limited our sample to include those who had previously accessed to the system. While our sampling procedure provided insight to veterans’ experiences using Secure Messaging, collecting data with those who did not register for access to use the Secure Messaging system would provide useful information about reasons for non-adoption. Third, this study included veterans without prejudice to their personal health history or absence thereof. Future research should explore Secure Messaging utilization, among those who may stand to benefit most, such as veterans with mental health problems or chronic health conditions. Fourth, this study did not capture the perspectives of staff or clinicians; these perspectives will be important to ascertain in future research, because study participants indicated that clinician resistance can impact patient use of Secure Messaging. This study and previous research [30] indicate that resistance on the part of clinicians can affect patient utilization. Understanding how clinicians’ perceptions of Secure Messaging influence patients’ adoption and use of this technology remains of interest for future study. Yet, previous work has also indicated clinicians’ perceived value of Secure Messaging, especially when becoming familiar with how it worked [7,28]. Fifth, we recognize that study participants may have altered how they use Secure Messaging after providing prospective informed consent for researchers to review their messages for the succeeding 3-month time period. Future studies may benefit from collecting retrospective message data, rather than prospective data, to ensure messages are not censored by users. In addition, though the content review was conducted for a 3-month timeframe, due to long periods of time when people do not routinely interact with their health care team, future studies should collect message data for a longer timeframe (eg, six months to one year).

### Conclusions

VA is invested in leveraging electronic health resources to facilitate patient-centered care for veterans and their families. Secure Messaging provides a patient-driven method of communication that can empower patients to effectively engage in continuous health relationships with their health care teams through meaningful use of this technology-based resource. As VA continues to promote Secure Messaging as a viable communication tool, results of this study can be used to improve and expand veterans’ use of Secure Messaging for better access to health care.

## Abbreviations

BRIEF: Brief Health Literacy Screening Tool
CEW: Computer-Email-Web Fluency Scale
eHEALS: eHealth Literacy Scale
PACT: Patient Aligned Care Teams
REALM: Rapid Estimate of Adult Literacy in Medicine Survey
TAM: Technology Acceptance Model
TPB: Theory of Planned Behavior
VA: Veterans Affairs
VAMC: VA Medical Center
